# On the Use of Ether and the Perchloride of Formyle, or Chloroform, in Surgical Operations

**Published:** 1848-01

**Authors:** 


### *On the use of Ether and the Perchloride of Formyle, or Chloraform, in Surgical Operations.*

We are indebted to Dr. S. G. Morton, for the following extract of a letter from Dr. James Suddards, of Philadelphia, dated Paris, December 1, 1847.

"In looking over the American papers a day or two since, I was somewhat surprised to read an article in the North American of our city, on the use of ether in surgical operations. The paper was from a gentleman in Boston, and recounted two cases of amputation, in which etherization had been employed with success; the wording, and indeed the whole tenor of his remarks, were such as to intimate rather an apology for a new discovery creeping into notice, than a description of what should now be considered an established therapeutical agent. It certainly is a matter of astonishment that in the very spot where its application for these purposes was first suggested, the surgeons and others, than whom there cannot be a more intelligent body in the world, should stand by, and doubtfully shake their heads, while the whole medical world of Europe should rise en masse, and loudly welcome the inventor as a benefactor of humanity. Is it not a shame to our country, and the profession, that we should calmly fold our hands before us, and allow our brethren in other parts of the world to reap the first fruits of a discovery which justly and rightly belongs to ourselves? To show the estimation in which it is held here, I need only mention that I have not seen a single operation during the whole five months of my residence, in which etherization was not employed and always with complete success; not an accident has happened, nor anything occurred to mar its happy effects. Indeed, so great is its reputation, that experiments have been made at the clinique of the faculty to see with what benefit it might be employed in labour; these operations I myself saw, and though they would scarcely justify an indiscriminate application, yet, as Dr. Smith of St. Bartholomew's, London, observes, they were so far beneficial as to lead us to hope great advantages, and stimulate to still further trial, if we might perchance lessen the pains of delivery, and do something for those who by nature and affection deserve so much at our hands. It would indeed, be considered cruel to subject a patient to the pain of any operation, however trivial in itself, when its avoidance could be purchased at so cheap a rate. I trust that surgeons in America have at last awakened to the benefits of an agent which has so long been waiting at their very doors, and are beginning, though late, to avail themselves of its happy effects. At the present moment there is a new discovery which, from all appearances, bids fair to rival the ether. This is the Perchloride of Formyle, first discovered by a German chemist, and afterwards obtained by Dumas, who elicited its characteristic properties.

In March last some experiments were made by Prof. Flourens, of Paris, who observed that when inhaled by small animals, insensibility was soon produced; but from some reason or other, his experiments were not continued, and it gradually faded from recollection. A few weeks since, it was again brought into notice by Drs. Simpson of Edinburgh, and Ferguson of London, who by its aid performed several operations, with little or no pain to the patients. One case was of a little boy four or five years of age, with necrosis of the tibia; a few drops being placed upon a handkerchief, and applied to the mouth and nostrils of the patient, insensibility was in a few moments produced, and the diseased bone removed by Professor Miller, without eliciting the least expression of suffering. I had the pleasure of seeing two experiments performed by Dr. Blandin of Hotel Dieu: the first was a woman, with an abscess on the thigh; the chloroform was administered in the manner recommended by Dr. Simpson, and insensibility produced in three and a half minutes; so that when the abscess was opened, not the least sensation was evident, nor indeed was the patient aware of the operation having been performed. In the second case, an operation for crushing stone in the bladder, (also a woman,) the application was made in the same manner as the ether, by an improved apparatus, invented by Luer. In this case, so great was the effect, that complete insensibility was produced in thirty seconds. If these cases can be considered as fair samples of the good effects of the new agent, it certainly may be regarded as a valuable acquisition to the surgeon. Its advantages over ether, according to Dr. Simpson, are as follows:—

1. The ease with which it may be applied, requiring no apparatus, but merely its application by handkerchief, as before mentioned.

2. It produces no coughing, irritation to the respiratory organs, or other unpleasant effect.

3. The rapidity of its action, requiring but one or two minutes at the utmost to produce complete insensibility."

